# Physical integrity and residual bio-efficacy of used LLINs in three cities of the South-West region of Cameroon 4 years after the first national mass-distribution campaign

**DOI:** 10.1186/s12936-017-1690-6

**Published:** 2017-01-17

**Authors:** Stravensky T. Boussougou-Sambe, Parfait Awono-Ambene, Geraud C. T. Tasse, Josiane Etang, Jerome A. Binyang, Lynda D. Nouage, Gaston Wamba, Peter Enyong, Eric B. Fokam

**Affiliations:** 1 Microbiology and Parasitology Department, University of Buea, P.O. Box 63, Buea, Cameroon; 2 Organisation de Coordination pour la lutte contre les Endémies en Afrique Centrale (OCEAC), B.P. 288, Yaoundé, Cameroun; 3 Laboratory for Biodiversity and Conservation Biology, University of Buea, P.O. Box 63, Buea, Cameroon; 4 Department of Zoology and Animal Physiology, University of Buea, P.O. Box 63, Buea, Cameroon; 5 Faculty of Medicine and Pharmaceutical Sciences, University of Douala, P.O. Box 2701, Douala, Cameroon; 6 Department of Biology and Animal Physiology, University of Douala, P.O. Box 2701, Douala, Cameroon; 7 Department of Biology and Animal Physiology, University of Yaoundé I, P.O. Box 3851, Yaoundé, Cameroon; 8 National Malaria Control Programme, South West Regional Delegation of Public Health, P.O. Box 281, Buea, Cameroon

## Abstract

**Background:**

Long-lasting insecticidal nets (LLINs) are effective tools at reducing malaria transmission. In Cameroon, following the first national mass distribution campaign in 2011, there has been no follow up on the efficacy of LLINs distributed. The aim of this report is to assess the physical integrity and insecticidal potency of LLINs distributed in three cities with contrasting socio economic status (SES) and to evaluate the use and care for bed nets owned by individuals.

**Methods:**

The study was conducted in Limbe, Tiko and Buea three localities in the Fako division of the South West Region of Cameroon. Tiko had the highest SES based on the type of building materials used, followed respectively by Limbe and Buea. The use and care for bed nets was assessed using a questionnaire, the physical state of bed nets evaluated following WHOPES recommendation for determining size of holes and the residual insecticidal activity of retrieved bed nets determined through a cone bioassay using susceptible strains of mosquitoes.

**Results:**

Of the 241 households visited in Limbe (n = 81), Tiko (n = 80) and Buea (n = 80), 186 (77.2%) had LLINs, with no significant variations from the selected study locations. However, bed net coverage was not meeting World Health Organization standards (p < 0.0001). Six different brands of LLINs were recorded, and the majority were those provided by the NMCP through the 2011 national mass campaign or antenatal care programme. Based on the calculation of the Proportionate Hole Index (PHI) as indicator of physical integrity of nets, the proportion of nets classified as serviceable (versus too torn) differed statistically according to locations (*p* value = 0.04), with 63.8% in Tiko, 50% in Limbe and 47% in Buea. Of the 20 nets tested for their efficacy against susceptible strains of mosquito, 42.6% (3 nets) were optimally effective in Tiko, 57.4% (4), 16.7% (1) and 14.3% (1) were minimally effective in Tiko, Buea and Limbe respectively. Finally; 85.7% (6) and 83.3% (5) were not effective in Limbe and Buea.

**Conclusions:**

These findings pinpoint the need for more frequent replacement of LLINs especially for people with low SES and also the need for the promotion of good practices on the maintenance and washing of nets.

## Background

Insecticide-treated nets (ITNs) have played an important role in the remarkable success in reducing malaria burden over the past decade with 69% of the 663 million fewer cases attributable to interventions due to ITNs [1]. The proportion of the population sleeping under an ITN has increased markedly in sub-Saharan Africa, from <2% in 2000 to an estimated 46% in 2014 and 55% in 2015 [1]. This increase comes as a result of the scale up of vector control using indoor residual spraying and delivery of long-lasting insecticidal nets (LLINs), with about 189 million delivered in 2014 and at least 154 million projected to be delivered in 2015 [1].

LLINs are factory-treated mosquito nets expected to retain their insecticidal activity for 20 standard World Health Organization (WHO) washes under laboratory conditions and at least 3 years of recommended use under field conditions [2]. They act as physical barriers thus preventing vector human contact and providing personal protection [3]. Their effectiveness is improved by the addition of insecticide and they still provide some protection even torn [4]. Many countries have embarked in mass LLINs distribution campaigns [5–10]. However little information exists on the physical durability of LLINs in the field; and few malaria control programmes to date have systematically monitored the performance of LLINs in terms of physical durability after mass distributions [10]. Aside for net fabric [11] and fibre weight or denier [12], other factors that influence physical deterioration are the house environment (house wall material, bed type and construction), socio economic status (SES) and the bed net maintenance behaviour (general handling, washing and repair) [13, 14].

Cameroon implemented a mass distribution of LLINs with almost 8,654,731 LLINs distributed throughout the country in 2011 [6]. Unfortunately, there was no follow up on the durability of the LLINs over the 4 years of their deployment. To monitor LLINs durability three elements need to be considered: survivorship, fabric integrity and bioefficacy [14]. Such information is extremely useful to determine the frequency at which these distribution campaigns have to be repeated in order to consolidate the gains of the intervention and significantly and durably curb the prevalence of malaria as the country is currently carrying out a second National distribution campaign. This is even more important as malaria vectors are developing resistance due to the scale up of control programmes and the use of pesticides for agro-industrial purposes. Here, the first assessment of physical integrity and insecticidal potency of LLINs distributed in three cities with contrasting socio economic **s**tatus of the South West Region of Cameroon is reported. Additionally, the use and care of bed nets in individual houses was also evaluated.

## Methods

### Study area and population

The study was conducted in Limbe (N 04°01′30.4″ E 009°11′40.5″), Tiko (N 04°04′32.6″ E 009°21′28.9″) and Buea (N 04°10′02.4″ E 009°18′27.3″), three localities in the Fako division of the South West Region of Cameroon. The area is subjected to a Cameroonian-type equatorial climate characterized by fairly constant temperatures and two seasons: a short dry season (November–February) and a long rainy season (March–October) with abundant precipitation (2000–10,000 mm) [15]. Temperatures are lower than in the other areas of the southern part of the country: the mean values of the minimum temperatures are 20 °C in December and 18 °C in August, the mean values of the maximum temperatures are 35 °C in August and 30 °C in March [16]. The total population in the Region was 1,316,079 inhabitants [17].

Interviews were carried out in selected quarters of the cities namely at the Bota CDC camp (Limbe) characterized by houses built with concrete materials and with most ceilings made up of paper, Holtforth (Tiko) characterized by houses built with concrete and most ceilings made up of plywood and finally in Muea (Buea) with most houses built with planks materials and most ceilings made up with paper. The houses were selected randomly.

### Bed net survey

Community-based, cross-sectional surveys were carried out in the three study sites using a questionnaire. Before administering the questionnaire, an informed consent was sought from the respondents. That consent was stating the purpose of the research and giving information on the study nets, on the type of study, on the right for participants to refuse or withdraw from the study and finally on the procedure.

The questionnaire was subdivided into three parts. The first was on personal and sociodemographic information of the respondents such as educational level of the head of the household, the number of people who slept in the household and the number of nets available to them. The second part was on the use and care for nets by asking for the source and age of the net, how often the net was used and on how and how often the net was washed. The third part was made up of questions to assess household building materials as an indicator of socioeconomic status (SES) in relation to the physical integrity of the nets. Interviews were not conducted in houses with no bed net; however, reasons for not having nets in such houses were recorded.

### Physical integrity of nets

The physical integrity of bed nets was assessed by checking for holes in the nets and counting them using the WHO Pesticide Evaluation Scheme (WHOPES) defined sizes as recommended by the WHO [18]:

- size 1: smaller than a thumb (0.5–2 cm),
- size 2: larger than a thumb but smaller than a fist (2–10 cm),
- size 3: larger than a fist but smaller than a head (10–25 cm) and
- size 4: larger than a head (>25 cm)

The total number of holes was categorized by size and position on the net (roof, upper, lower and seams) and recorded.

### WHO cone bioassays

Ten nets were withdrawn per city and replaced with new LLINs. Five pieces were cut from each net, wrapped in aluminum foil and kept at 4 °C until insecticide activity tests were performed. The tests were carried out at the Entomology Laboratory of “Organisation de Coordination pour la lutte contre les Endémies en Afrique Centrale” (OCEAC, Yaoundé, Cameroon). The Kisumu (*Anopheles gambiae*) and Ngousso (*Anopheles coluzzii*) susceptible *Anopheles* strains were used alternatively as controls depending on their availability. The Ngousso colony has been established in January 2006 from larvae collected in Ngousso, a suburb of Yaounde and reared at the OCEAC insectary under standard conditions. The Ngoussou colony is a fully DDT-susceptible colony with no known *kdr* mutations [19]. Cone bioassays were performed according to the WHO protocol [18]; 4 cones were fixed to each piece of the net and 5–10 mosquitoes transferred in each cones. Mosquitoes were exposed in plastic cones for 3 min and transferred to observation cups. Knock down was recorded for 60 min after exposure. The mosquitoes were then fed a 10% sugar solution on soaked cotton balls and mortality was recorded for 24 h after exposure.

### Data analysis

The hole counts were used to calculate the Proportionate Hole Index (pHI) for each net by weighting each hole by size and summing them for each net using the following formula by WHO [14]:

- pHI = (1 × no. of size-1 holes) + (23 × no. of size-2 holes) + (196 × no. of size-3 holes) + (578 × no. size-4 holes)

The holes were weighted according to the average area of each hole category. For the hole size categories described above, the weights were 1, 23, 196 and 578, which corresponded to the areas estimated on the assumption that the hole sizes in each category are equal to the mid-points.

The pHI was then used to classify the nets in different categories following recommendations by WHO [20] into good, damaged and too torn nets. Those with pHI between 0 and 64 were considered as being in “good” condition where there is no reduction of efficacy compared to an undamaged net; those with pHI between 65 and 642 were considered in “acceptable” condition in the sense that their effectiveness is somewhat reduced but still provide significantly more protection than no net at all; and finally those “too torn” with pHI equal or >643 where the protective efficacy for the user is in serious doubt and the net should be replaced as soon as possible. Also, another category namely “serviceable” was considered and was made up of the categories of “good” and “damaged” nets.

The bioassay results for the netting pieces of nets from each sampled LN were pooled to determine if the net meets the WHO efficacy requirement:

- For optimal effectiveness: ≥80% mortality or ≥95% knockdown [2]
- For minimal effectiveness: ≥50% mortality or ≥75% knockdown [19]
- Not effective: <50% mortality or <75% knockdown

Data collected were entered in Microsoft Excel 2013 and graphs were drawn. The R software version 3.2.5 [21] was used to compare the use and care of nets between the three cities using the Chi squared test, Student t test, Kruskal–Wallis. Additionally a post hoc analysis was performed using the Dunnet test to make a pair wise comparison of mean Hole Indexes between the three cities. Tests were considered statistically significant for P values <0.05.

### Ethics, consent and permissions

Before commencement of the study, administrative clearance was obtained from the South West Regional Delegation of Public Health. The Institutional Review Board of the University of Buea issued an ethical clearance. Additional authorizations were obtained from the local health authorities in Limbe, Tiko and Buea. Also at the beginning of the data collection exercise, chiefs of the study communities were met and their consent for the study obtained. Before administering the questionnaire, a signed informed consent was obtained from the respondents

## Results

### LLINs coverage, sources and age

A total of 241 adults were interviewed in Limbe, Tiko and Buea during the study. Out of these, 186 (77.2%) had bed nets. In Limbe, Tiko and Buea, respectively, 77.5% (62 out of 81), 82.5% (66 out of 80) and 72.5% (58 out of 80) households visited had at least one LLIN. The others citing various reasons for not having them, including that: nets were either too torn, old or that they did not receive any during the previous campaign. There was a significant difference (t = 10.679, p < 0.0001) between the observed ratio of nets per persons (2.1 ± 1.2) and the recommended number of nets (one net for every two persons set by the WHO (3.1 ± 1.4). Out of the households visited 36 (44.4%) in Limbe, 29 (36.3%) in Tiko and 38 (47.5%) in Buea had the recommended number of nets. There was no statistical difference between the three cities regarding the proportion of houses with the recommended number of nets (χ^2^ = 2.214, df = 2, p value = 0.3306). All the nets recorded were LLINs of different brands, with the most common being Olyset nets (77.4%) followed by Permanet 2.0 (15.1%), Siamdutch (4.9%), Yorkool (1.6%), Dawa (0.5%) and Safi net (0.5%).

Of the nets inspected, 71, 74.1, 80.3% in Limbe, Tiko and Buea respectively were from the 2011 mass campaign distribution of the National Malaria Control Programme (NMCP). Additionally, 19.4% (Limbe), 22.4% (Tiko) and 10.6% (Buea) of the respondents received their mosquito nets through AnteNatal Care (ANC) with the remaining reporting to have bought their nets. Most respondents in the three cities had reportedly used their nets for 3–4 years 64.5% in Limbe, 68.2% in Tiko and 60.3% in Buea. There was no difference between the three cities with regards to the age of the nets (N = 186; χ^2^ = 7.4703; df = 6; P = 0.27).

### Use and care of nets

One hundred and seventy-five respondents (94.1%) reported that they had used a net the previous night. Out of the 186 respondents, 178 of them (95.7%) reported that they had used a net every night (7 nights) and 179 of them (96.2%) reported that they use a net all year round. Most respondents reported that they had washed their nets at least once, 74.2% in Limbe, 72.4% in Tiko and 74.2% in Buea. There was no statistical difference between the three cities regarding the proportion of nets washed (χ^2^ = 4.3245, df = 4, p value = 0.3639).

To assess if the respondents were following recommendations set by the NMCP i.e. to use bar soap and to dry the nets under the shade, respondents were asked about the types of soaps used to wash the nets. Most of them in the three cities used different types of soap with the most common being detergent (41%), a mix of bar soap and detergent (34%) and local bar soap (20%). Most of the nets in the three cities (88.4%) were dried outside in the sun while only few (10.1%) of them were dried outside in the shade.

### Bed nets physical integrity

**Fig. 1:**
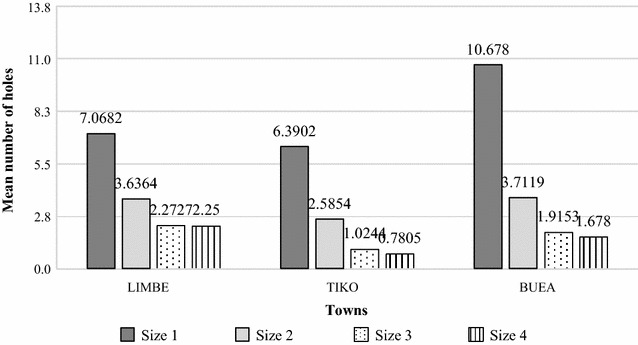
Mean number of holes per category size in each city

Out of the 186 nets inspected for presence of holes, most of the nets in the three cities had at least one hole: 89.4, 70.7 and 71% in Buea, Tiko and Limbe, respectively (Table 1). In total, 2173 holes were counted, most of them (92%) located on the lower parts of the nets. All the four categories of holes were found, of which small holes (size 1) were the most common making up to 55.4% followed by those of size 2 (22.3%), size 3 (11.7%) and size 4 (10.6%), respectively. There was a significant statistical difference in the number of holes counted between the three cities (χ^2^ = 42.735, df = 6, p < 0.0001) with Buea having the nets with the highest number mean number of holes per each hole category size, followed by Limbe and Tiko, respectively (Fig. 1).

**Table 1:** Mean Proportionate Hole Index estimated in each locality

	Number of nets assessed	Number (%) of nets with holes	Mean pHI*
Limbe	62	44 (71.0)	1493.1^a, b^
Tiko	58	41 (70.7)	723.7^b^
Buea	66	59 (89.4)	1603.2^a^

Asterisk indicate values with different letter superscript are significantly different

There was a significant statistical difference (Kruskal–Wallis χ^2^ = 7.969, df = 2, p value = 0.0186) in the mean Proportionate Hole Index (pHI) of nets in the three cities with Buea having nets with the highest mean pHI and Tiko having the lowest. There was a significant statistical difference between Tiko and Buea and none between Tiko and Limbe, however there was no significant statistical difference between Limbe and Buea.

The pHI ranged from 0 to 8700 and was used to categorize the nets into three categories good, damaged and too torn. In Limbe, out of the 62 nets inspected, 31 nets (50.00%) were considered as too torn, 27 (43.5%) had a pHI lower than 64 and were considered as good nets while the remaining 4 nets (6.5%) were found to be damaged.

In Tiko, 29 nets (50%) out of the 58 inspected for holes were considered as good. Eight nets (13.8%) were found to be damaged and the remaining 21 nets (36.2%) were found to be too torn. In Buea, 35 nets (53%) out of the 66 nets inspected for holes were found to be too torn, 18 nets (27.3%) were found to be good while the remaining 13 (19.7%) were considered to be damaged.

**Fig. 2:**
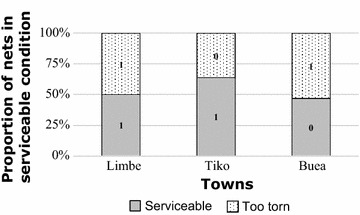
Proportion of serviceable (good + damaged) and too torn nets in Limbe, Tiko and Buea

The pHI was also used to classify the nets into two categories those that are serviceable and those that are too torn (Fig. 2). The highest proportion of nets in serviceable condition (63.8%) was found in Tiko followed by Limbe (50%). In Buea, where the highest number of nets with holes (89.4%) was recorded, the lowest proportion (47%) of serviceable nets was also found (Fig. 2). There was a significant statistical difference in the physical integrity of LLINs between the three cities (χ^2^ = 6.4634, df = 2, p = 0.03949).

### Bed nets bioassays

The insecticidal activity of bed nets was determined using the Kisumu and the Ngousso susceptible strains. Twenty out of the 30 nets collected were tested and using KD60 and mortality of the susceptible strains, they were classified as nets with optimal effectiveness, minimal effectiveness and those not effective. 1331 mosquitoes were used to test the nets in Limbe. Out of the 7 nets tested only one met the requirement for minimal effectiveness with a mortality of 60%, the rest of the nets (85.7%) were not effective as mortalities were below 50% (Table 2).

**Table 2:** Residual bioefficacy of used nets in Limbe, Tiko and Buea

Sites	Brand	No.	KD 60 (±5% CI)	Mortality (±5% CI)	Status
Limbe	Permanet 2.0	192	0.5% (±0.1)	4.1% (±2.8)	Not effective
	Olyset net	199	1% (± 1.3)	3% (±2.4)	Not effective
	Olyset net	197	4.6% (±2.9)	2.5% (±2.2)	Not effective
	Siamdutch	189	29.1% (±6.5)	10.1% (±4.3)	Not effective
	Siamdutch	192	13.5% (±4.8)	29.1% (±6.4)	Not effective
	Olyset net	177	29.3% (±6.7)	60% (±7.2)	Minimally effective
	Olyset net	185	3.2% (±2.5)	3.9% (±2.8)	Not effective
Tiko	Olyset net	103	52.4% (±9.6)	74.8% (8.4)	Minimally effective
	Permanet 2.0	115	84.3% (±6.6)	82.6% (6.9)	Optimally effective
	Siamdutch	88	60.3% (±10.2)	54.5% (±10.4)	Minimally effective
	Olyset net	117	89.7% (±5.5)	81.2% (±7.1)	Optimally effective
	Olyset net	117	75.2% (±7.8)	55.6% (±9.0)	Minimally effective
	Olyset net	99	93.9% (±4.7)	71.7% (±8.9)	Minimally effective
	Olyset net	115	91.3% (±5.2)	83.5% (±6.8)	Optimally effective
Buea	Olyset net	189	4.2% (±2.9)	14.8% (±5.1)	Not effective
	Olyset net	138	20.3% (±6.7)	17.4% (±6.3)	Not effective
	Olyset net	217	11.5% (±4.2)	14.3% (±4.7)	Not effective
	Permanet 2.0	178	4.5% (±3.0)	5.6% (±3.4)	Not effective
	Olyset net	178	3.4% (±2.7)	4.5% (±3.0)	Not effective
	Permanet 2.0	107	93.5% (±4.7)	75.7% (±8.1)	Minimally effective

No: Number of mosquitoes used to test the net

In Tiko, 7 nets were tested using 754 mosquitoes. Out of the 7 nets tested, 3 met the requirements for optimal effectiveness (42.6%), while the remaining 4 (57.4%) had a minimal insecticidal effectiveness (Table 2). In Buea, 1007 female mosquitoes were used to test the nets. Out of the 6 nets tested 1 (16.7%) met the requirement for minimal effectiveness with a mortality of 51% while the remaining 5 (83.3%) were found to not be effective (Table 2).

## Discussion

This study assessed the physical integrity and insecticidal potency of bed nets distributed in three cities of South West Region of Cameroun 4 years after the first mass-distribution campaign of bed nets in 2011. Additionally, the use and care for bed nets was evaluated. LLINs ownership was high in the three cities reaching up to 82.5% in Buea. The sustainment of such a high ownership 4 years after a nationwide distribution campaign can be explained by the fact that people were able to get access to nets through ANC or by simply buying them and also to the awareness of the importance of bed nets. Some people had kept surplus bed nets from the previous distribution campaign which were then used to replace old nets. The same high ownership was observed by Sumbele et al. [22] among children in Muea and by the Ministry of Public Health [22] in the South-West Region. A significant difference was observed between the standard set by the WHO of one net for every two persons and the net coverage on sites as also found by the Ministry of Public Health [23], with most households in the three cities not having the recommended number of nets; this means that the other means of provisioning of nets were not able to make up for the nets lost over the years after the distribution. All the inspected nets were LLINs, with Olyset being the most commonly used (77.42%) used followed by Permanet 2.0. These two brands of LLINs were the ones distributed by the NMCP during the 2011 campaign [6]. The durability of Olyset Net^®^ as advised by the manufacturer is 5 years (minimum) from the first use. The majority of nets found in this study were within the 36–48 old months range but there was no statistical differences between the ages of nets in the three cities. The second source of nets was from ANC followed by those that were purchased in the three cities.

The proportion of nets with holes was high, reaching 89.39% in Buea; this was expected as it has been shown in Uganda that considerable physical damage (45–78% of damaged nets) can occur on bed nets even within a year of bed net use in operational conditions [13, 24]. Most of the holes were located on the lower part of the nets which is the part tucked under the mattress which often get caught on parts of the bed in the course of handling.

Factors that influence physical deterioration are the house environment (house wall material, bed type and construction), socio economic status (SES) and the bed net maintenance behaviour (general handling, washing and repair) [13, 14]. Buea had the highest proportion of nets with holes (89.4%) with Limbe and Tiko having almost the same proportions 71 and 70.7% respectively. The houses visited in the three cities were mostly built using different materials with households visited in Limbe and Tiko built in concrete, while those in Buea were built with plank material which could be an explanation for the high number of holes recorded in this area as house wall material could influence physical deterioration [13, 14]. The WHO classification allowed classification of nets into three categories which were Good, Damaged and Too torn [19] using the pHI calculated for each net. Tiko had the highest proportion of nets in good condition followed by Limbe, those nets are considered as nets with no reduction of efficacy when compared to undamaged nets. In Buea, the majority of nets were found to be too torn, here the type of building material could play a role as Buea where the highest number of nets with holes was also the area with the most deteriorated nets. The use of the other two categories namely serviceable and too torn, allowed us to identify households that have to be prioritized for nets replacement. The highest proportion of nets that needed replacement was found in Buea with 53% of them that were too torn and whose protective efficacy for the user was in serious doubt. In Limbe, half of the nets needed to be replaced, while in Tiko only 36.2% of the nets needed immediate replacement. There was a statistical difference between mean Hole Indexes of bed nets between the three towns although no statistical significance was found when they were compared pair wise which may be due to the low sample size. This points to the presence of underlying factors causing nets to lose their physical integrity faster in Buea as compared to the other communities.

There was difference in ceiling materials with most ceilings in Tiko made with plywood while in Limbe there were mostly made up of paper, hence ceiling materials may also play a role in nets deterioration. These results allowed the identification of wall and ceiling materials as predictors for poor physical condition of bed nets owned by individuals from areas in the South West Region of Cameroon. Household building materials which can also be used to assess the socio-economic status (SES) of populations [25] show that nets tend to be more deteriorated in poorer communities such as Buea as compared to areas with individuals with a higher SES such as those found in cities such as Limbe and Tiko. Similar results were also found by Mutuku et al. [9] with nets from higher SES families found in significantly better physical condition compared to those from lower SES families. Therefore, poor communities have to be prioritized for nets replacement by the NMCP as there is emerging evidence that any protection added by the pyrethroid in damaged nets is lost if the vectors are resistant as demonstrated in areas of Benin, Kenya, Burkina-Faso and Cameroon [4, 26–29], thus compromising the effectiveness of malaria control efforts.

The bioassays were done using the Kisumu (*A. gambiae*) and Ngousso (*A. coluzzii*) strains as both species occur in the study areas. The results of the bioassays followed the same trend as that of physical integrity, with nets with the most effective insecticidal activity found in Tiko, where all the nets were effective. Majority of nets in Limbe and Buea were not effective. The maintenance of the insecticidal effectiveness may be influenced by type of building materials of homes where they are deployed and therefore by SES of communities. Tiko was the community with the highest SES based on building materials; it can be assumed that under such conditions, nets are exposed to conditions that prevent rapid physical deterioration and loss of insecticide potency as quickly as in poorer communities. There was low compliance to manufacturer’s recommendations on how the nets should be washed and dried, even though it might play a role in the loss of insecticidal effectiveness it was difficult to link it with the loss of effectiveness here as LLINs in Tiko were still effective despite going through similar treatments.

Despite of the age of the nets, reported use rate was very high with about 94.1% of the respondents reporting that they had used a net the previous night; the same was observed for the frequency of net use and this reported high use rate is in accordance with previous findings [20]. A drop in net usage as they were getting old and holed would have been expected as shown by previous studies [5, 9] which indicated that the number of bed nets used decreased with increasing bed net age for bed net 0–3 year old. In addition, the fact that no seasonality in the use of LLINs was observed is another indication of high compliance by local populations to prescribed guidelines; this may be due to the perennial transmission of malaria in this part of the country. Moreover, this high use of LLINs observed among populations all year round may be accounted for by the presence of permanent larval developmental spots such as gutters and water containers, that maintain year-round nuisance of mosquitoes as also observed by Ossè et al. [30] in Benin. However, these results are to be put in perspective as from the data collected, there is a clear indication that people were answering the question on reported net usage for convenience as most of the nets in Buea and Limbe for example had obviously lost their physical integrity and insecticidal potency, thus affording little or no protection and putting their usefulness in serious doubt.

There were some potential limitations in this study: firstly, as reported by Mutuku et al. [9], the source of nets and whether/how they were used, their age, frequency of washing and type of soap used were all based on self-reports, which often is subject to respondent bias. Secondly the fact that this was a cross sectional study did not allow for evaluation of net survivorship and attrition. Thirdly as this study was carried out 4 years post-deployment there was no follow-up information on the gradual performance of nets over the years.

## Conclusion

This study is the first of its kind to report on the performance of nets under operational conditions in Cameroon by checking the physical integrity and the insecticidal potency of nets post deployment. These findings are significant for NMCPs as they permitted identification of poor communities as priority areas for nets replacement following national distribution campaigns as most nets in Buea had lost their physical integrity and insecticidal capacity and offered little to no protection against malaria.

## Abbreviations

ANC: antenatal care
BUCREP: Bureau Central des Recensements et des Etudes de Population
CDC: cameroon development corporation
ITNs: insecticide-treated nets
LLIN: long-lasting insecticidal net
OCEAC: Organisation de Coordination pour la lutte contre les Endémies en Afrique Centrale
NMCP: National Malaria Control Programme
pHI: Proportionate Hole Index
SES: socio economic status
WHO: World Health Organization
WHOPES: World Health Organization Pesticide Evaluation Scheme
